# In Vitro Assessment of the Antiviral Activity of Ketotifen, Indomethacin and Naproxen, Alone and in Combination, against SARS-CoV-2

**DOI:** 10.3390/v13040558

**Published:** 2021-03-26

**Authors:** Pantea Kiani, Andrew Scholey, Thomas A. Dahl, Lauren McMann, Jacqueline M. Iversen, Joris C. Verster

**Affiliations:** 1 Division of Pharmacology, Utrecht Institute for Pharmaceutical Sciences (UIPS), Utrecht University, 3584 CG Utrecht, The Netherlands; p.kiani@uu.nl (P.K.); j.c.verster@uu.nl (J.C.V.); 2 Centre for Human Psychopharmacology, Swinburne University, Melbourne, VIC 3122, Australia; andrew@scholeylab.com; 3 Sen-Jam Pharmaceutical, 223 Wall St., #130, Huntington, NY 11743, USA; tadahl@sen-jam.com (T.A.D.); lmcmann@sen-jam.com (L.M.)

**Keywords:** antiviral, drug repurposing, SARS-CoV-2, COVID-19, SJP-002C, indomethacin, ketotifen, naproxen, mast cell stabilizer

## Abstract

The 2019 coronavirus infectious disease (COVID-19) is caused by infection with the new severe acute respiratory syndrome coronavirus (SARS-CoV-2). Currently, the treatment options for COVID-19 are limited. The purpose of the experiments presented here was to investigate the effectiveness of ketotifen, naproxen and indomethacin, alone or in combination, in reducing SARS-CoV-2 replication. In addition, the cytotoxicity of the drugs was evaluated. The findings showed that the combination of ketotifen with indomethacin (SJP-002C) or naproxen both reduce viral yield. Compared to ketotifen alone (60% inhibition at EC_50_), an increase in percentage inhibition of SARS-CoV-2 to 79%, 83% and 93% was found when co-administered with 25, 50 and 100 μM indomethacin, respectively. Compared to ketotifen alone, an increase in percentage inhibition of SARS-CoV-2 to 68%, 68% and 92% was found when co-administered with 25, 50 and 100 μM naproxen, respectively. For both drug combinations the observations suggest an additive or synergistic effect, compared to administering the drugs alone. No cytotoxic effects were observed for the administered dosages of ketotifen, naproxen, and indomethacin. Further research is warranted to investigate the efficacy of the combination of ketotifen with indomethacin (SJP-002C) or naproxen in the treatment of SARS-CoV-2 infection in humans.

## 1. Introduction

Coronaviruses are a large family of viruses that cause illness ranging from the common cold to more serious diseases such as Middle East Respiratory Syndrome (MERS) and Severe Acute Respiratory Syndrome (SARS). In December 2019, an outbreak of respiratory disease in China was found to be caused by a novel coronavirus SARS-CoV-2 that causes the disease Coronavirus Infectious Disease of 2019 (COVID-19).

**Figure 1:**
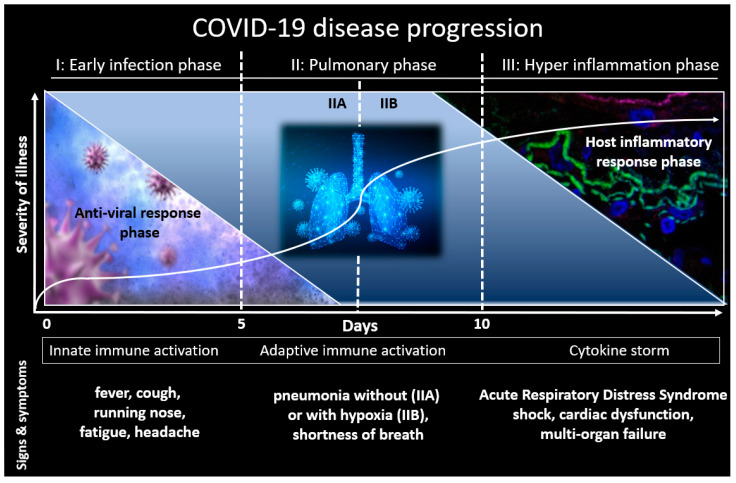
COVID-19 disease progression.

SARS-CoV-2 virus is an enveloped, positive-sense, single-stranded RNA beta-coronavirus [1,2,3,4,5,6]. The clinical progression of COVID-19 ranges from asymptomatic carriage to fulminant cytokine storm with respiratory failure, multi-organ dysfunction and ultimately death. It has been described that SARS-CoV-2 mediated inflammatory response has three different stages [5] (see Figure 1). Stage I, early infection, is characterized by increased cytokine and chemokine production. Stage I is the incubation period when SARS-CoV-2 multiplies and establishes residence in the host, primarily focusing on the respiratory system. Symptoms reported during Stage I are usually mild and often non-specific, including fever, cough, diarrhea, and headache. Stage II of COVID-19 infection, the pulmonary phase, is divided into two distinct parts: pneumonia without hypoxia (Stage IIA) or pneumonia with hypoxia (Stage IIB). During Stage IIB, symptoms worsen and patients will likely require hospitalization and oxygen supplementation. Stage III is the most severe stage, which manifests as an extrapulmonary systemic hyper inflammation syndrome, driven by the cytokine storm. Stage III is characterized by exuberant inflammation resulting from high circulating cytokines such as interleukin (IL)-6 and tumor necrosis factor alpha (TNF-α) [7,8,9,10,11,12]. These inflammatory mediators are responsible for the multi-organ damage, including acute respiratory distress syndrome (ARDS) and cardiac and renal dysfunction [13,14,15]. In general, the prognosis and recovery from Stage III are poor.

In addition to inhibition of viral replication, blunting this inflammatory response before overt cytokine storm is important to improve outcomes [7,16,17]. Although remdesivir and convalescent plasma are used for this purpose, there is a need for adjunct treatments to ameliorate disease burden.

In vitro susceptibility of viruses to an antiviral agent is usually assessed using a quantitative assay to measure virus replication in the presence of increasing concentrations of the product compared to replication in the absence of the product. The half maximal effective concentration is the concentration of product at which virus replication is inhibited by 50 percent (EC_50_). Assays that evaluate antiviral activity include, but are not limited to, virus inactivation assays, plaque reduction assays, cytopathic effect inhibition assays, peripheral blood mononuclear cell (PBMC) assays, and binding and fusion assays [18]. Additionally, compounds must also be evaluated for cellular toxicity. The cytotoxic effect of a compound is usually determined in a cell line of relevance, by calculating the cellular cytotoxicity concentration (CC_50_), i.e., the concentration of the test compound that reduces cell viability by 50%, compared with the cell control value.

Data of animal models and non-human primate models support that mast cells are strongly activated during SARS-CoV-2 infection, producing damage to the lung tissue, with haemorrhaging visible on the lungs and fluid accumulation in the lungs [19,20]. Traditionally, mast cell stabilizers such as ketotifen have been in used in humans for the treatment of allergy and asthma, and these drugs are thought to act by preventing degranulation of mast cells [21,22,23]. Sen-Jam Pharmaceutical is developing combination therapeutics for the treatment of COVID-19, including a mast cell stabilizer and a non-steroid anti-inflammatory drug. These products are thought to have both antiviral and anti-inflammatory properties, capable of reducing viral replication and the excessive immune response, typically referred to as cytokine storm. In the current study, the combination product ketotifen and indomethacin (SJP-002C) and the combination product ketotifen and naproxen are investigated. It is hypothesized that both combination products can be used early in the treatment of COVID-19 to modify disease progression, and in combination with standard of care. As broad-spectrum anti-inflammatory drugs, reducing both cyclooxygenase and inhibiting mast cell degranulation, together ketotifen and indomethacin can maximally reduce immune mediated inflammation. A further advantage of the combination products is that ketotifen may provide protection against possible indomethacin or naproxen-induced gastrointestinal (GI) injury.

The purpose of the experiments presented here were to investigate the effectiveness of ketotifen, naproxen and indomethacin, alone or in combination, in reducing virus replication. In addition, the cytotoxicity of the drugs was evaluated.

## 2. Materials and Methods

Ketotifen, indomethacin, and naproxen were sourced from Sigma Aldrich and remdesivir from MedChemExpress SARS-CoV-2 hCoV-19/Australia/VIC01/2020 was a gift from Melbourne’s Peter Doherty Institute for Infection and Immunity and African Green Monkey Kidney (Vero E6) cells obtained from ATCC (ATCC-CRL1586) (Noble Park North, VIC, Australia).

Virus stocks were expanded via passage in Vero E6 cells in growth media, which comprised Minimal Essential Medium without l-glutamine supplemented with 1% (*w*/*v*) l-glutamine 1.0 μg/mL of TPCK-Trypsin, 0.2% BSA, 1× Pen/Strep, and 1% Insulin Transferrin Selenium (ITS).

African Green Monkey Kidney (Vero E6) cells (ATCC-CRL1586) were sub-cultured to generate cell bank stocks in cell growth medium, which comprised of Minimal Essential Medium (MEM) without l-glutamine supplemented with 10% (*v*/*v*) heat-inactivated Fetal Bovine Serum (FBS) and 1% (*w*/*v*) l-glutamine. Vero E6 cells were passaged for a maximum of 13 passages, after which a new working cell bank stock was retrieved from liquid nitrogen for further use.

### 2.1. Study 1. Cytopathic Effect (CPE) Assay

In Study 1, the cytopathic effects of indomethacin, ketotifen, and naproxen were compared with remdesivir (positive control).

Vero E6 cells were seeded into 96-well plates at 2 × 10^4^ cells/well in 100 μL seeding medium (MEM supplemented with 1% (*w*/*v*) l-glutamine, 2% FBS). Plates were incubated overnight at 37 °C, 5% CO_2_. Test compounds were prepared fresh on the day of testing, vortexed and visually inspected to confirm complete dissolution.

The positive control compound remdesivir was prepared as a 10 mM stock in dimethyl sulfoxide (DMSO) and stored at −20 °C. Compound dilutions were prepared on the day of experimentation. A 3-fold, 8 point, DMSO dilution series of each of the four test compounds was performed initially, ranging 100 mM to 0.045 mM. An intermediate dilution series in virus growth medium (MEM supplemented with 1% (*w*/*v*) l-glutamine, 2% FBS, 8 μg/mL Tosyl Phenylalanyl Chloromethyl Ketone (TPCK)-Trypsin was generated ranging 800 µM–0.37 µM. A 50 μL volume from each compound intermediate dilution series was added to triplicate wells of the assay plate pre-seeded with Vero E6 cells. The DMSO concentration was maintained at 0.2% in the assay plate. One assay plate per compound was generated.

Similarly, remdesivir was subjected to an initial DMSO dilution series, intermediate dilution series to reduce the DMSO concentration to 0.2% in the assay plate. Remdesivir was tested at a starting concentration of 20 µM.

A 50 μL volume of SARS-CoV-2 diluted in virus growth medium to generate a multiplicity of infection (moi) of 0.05, was added to the assay plates. This moi was previously determined to provide 100% cytopathic effect (CPE) in 4 days. Virus was added to triplicate rows to assess antiviral activity and virus growth medium without virus was added to triplicate rows to assess cytotoxicity.

The percent cell protection achieved by the positive control (remdesivir) and test articles in virus-infected cells was calculated by the formula of Pauwels et al. [24] as shown below:

Percent cell protection = ([ODt]virus − [ODc]virus/[ODc]mock − [ODc]virus) × 100(1)

where:

[ODt]virus = the optical density measured in a well examining the effect of a given concentration of test article or positive control on virus-infected cells.

[ODc]virus = the optical density measured in a well examining the effect of the negative control on virus-infected cells.

[ODc]mock = the optical density measured in a well examining the effect of the negative control on mock-infected cells.

The EC_50_ values were calculated from the percent cell protection results by non-linear regression analysis using the Hill (sigmoid Emax) formula:

y = Min_y_ + (Max_y_ − Min_y_)/1 + (EC_50_/x)^D^ (2)

where:

X = test or control article concentration;
Y = percent cell protection;
Min = minimum;
Max = maximum;
D = slope coefficient.

### 2.2. Study 2. Yield Reduction Assay

Vero E6 cells were seeded into 96-well plates at 2 × 10^4^ cells/well in 100 μL seeding medium (MEM supplemented with 1% (*w*/*v*) l-glutamine, 2% FBS). Plates were incubated overnight at 37 °C, 5% CO_2_. Ketotifen, indomethacin and naproxen were prepared as outlined in Section 2.1 above.

For combination studies, compounds were added together in the deep well plate in a 1:1 ratio, then 100 μL volume of compound combination was added to cell monolayers. A 200 μL volume of virus (B3) was added to plates (moi 0.003).

After 48 h incubation, virus was harvested at each concentration and diluted 1:10 (*n* = 1) or 1:100 (*n* = 2) in virus growth media. One hundred microliters were added to triplicate wells of 96-well plates containing Vero E6 cells and serially diluted three-fold across the plate for a total of nine different virus concentrations. Six of the wells contained assay media alone (i.e., no virus) and served as controls. Plates were incubated for three days at 37 °C in a humidified 5% CO_2_ atmosphere during which time the CPE was allowed to develop. Cell monolayers were then observed microscopically with visual scoring of virus-induced CPE used as an endpoint. The TCID_50_ of the virus suspension was determined using the method of Reed-Muench [25]. The virus yield was expressed as a percentage with respect to virus growth when no drug was added, for each concentration.

### 2.3. Study 3. Cytotoxicity Assay

The cytotoxic effects of indomethacin, ketotifen, and naproxen on Vero E6 cells was tested over a 4 day or 48 h period to mimic either the antiviral assay or the yield reduction assay. Drugs were tested at the same concentration as those used in Section 2.1 and Section 2.2. Viable cells were determined by staining with MTT or crystal violet. A 100 μL volume of a 3 mg/mL solution of MTT was added to plates and incubated for 2 h at 37 °C in a 5% CO_2_ incubator. Wells were aspirated to dryness and formazan crystals solubilized by the addition of 200 μL 100% 2-Propanol at room temperature for 30 min. Absorbance was measured at 540–650 nm on a plate reader. Media were removed from cells stained with crystal violet and washed once with PBS. A 40 μL volume of 0.25% crystal violet/20% methanol stain was added to each well and incubated for 30 min at room temperature. Crystal violet stain was aspirated, monolayers washed 3–5 times with PBS and the stain solubilized with the addition of 100 μL 1% sodium dodecyl sulfate (SDS). After 30 min at room temperature, absorbance was measured at 540–650 nm on a plate reader. The 50% cytotoxic concentration (CC_50_) was defined as the concentration of the test compound that reduces the absorbance of the mock infected cells by 50% of the control value. The CC_50_ value was calculated as follows:

CC_50_ = [ODt]mock/[ODc]mock. (3)

## 3. Results

### 3.1. Study 1. Cytopathic Effect (CPE) Assay

Results of Study 1 are shown in Table 1. None of the treatments exhibited antiviral activity in the 4 days CPE assay.

### 3.2. Study 2. Yield Reduction Assay

**Figure 2:**
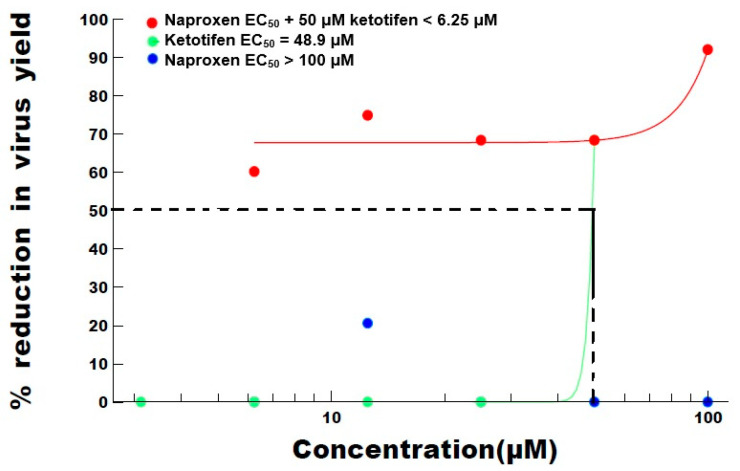
Dose–response curves for naproxen and ketotifen, alone and in combination. EC_50_ is the effective concentration of product, i.e., the concentration at which virus infection is inhibited by 50 percent.

The compounds were then tested in a Yield Reduction Assay (YRA) under reduced rounds of virus replication (48 h) to see if any antiviral activity was observed. The results for ketotifen and naproxen are summarized in Table 2 and Figure 2. Ketotifen alone inhibited SARS-CoV-2 and exhibited an EC_50_ of 48.9 μM. Naproxen alone did not inhibit SARS-CoV-2 and exhibited an EC_50_ of >100 μM.

When ketotifen was added at a single concentration, naproxen’s ability to reduce virus yield was enhanced. The EC_50_ of naproxen was reduced to <6 μM, equating to ~17-fold greater effect in the presence of ketotifen. The dose–response curves are shown in Figure 2. These data indicate an additive or synergistic effect (See Figure 2).

The results for ketotifen and indomethacin are summarized in Table 3 and Table 4. Table 3 reveals that, compared to ketotifen alone (60% inhibition at EC_50_), an increase in percentage inhibition of SARS-CoV-2 to 79%, 83% and 93% was observed when co-administered with 25, 50 and 100 μM indomethacin, respectively, indicating an additive or synergistic effect.

Indomethacin alone inhibited SARS-CoV-2 and exhibited an EC_50_ of 100.1 μM (see Table 4). Indomethacin did not reach an EC_50_ in the presence of 50 μM ketotifen, although 60% inhibition was observed at 6.25 μM indicating that an EC_50_ would probably be reached at ~3 μM had the dilution series had covered a lower range. These in vitro data indicate that a lower dose of indomethacin is required to inhibit virus growth in the presence of 50 μM of ketotifen, indicating an additive or synergistic effect. Indomethacin did not reach an EC_50_ in the presence of 25 μM, 12.5, 6.25 or 3.1 μM ketotifen.

**Figure 3:**
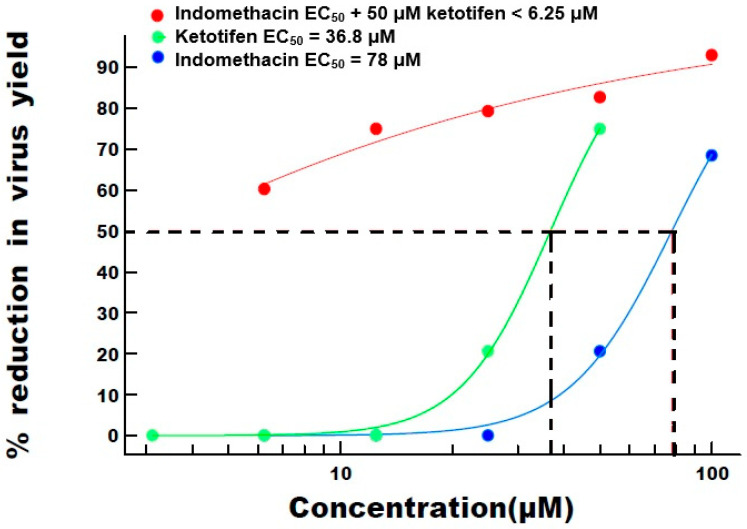
Dose–response curves for indomethacin and ketotifen, alone and in combination.

When ketotifen was added at a single concentration, indomethacin’s ability to reduce virus yield was enhanced. The EC_50_ of indomethacin was reduced to <6 μM, equating to ~13-fold greater effect in the presence of ketotifen. Figure 3 shows the dose–response curves for indomethacin and ketotifen, alone and in combination. The dose–response curves suggest an additive or synergistic antiviral effect against SARS-CoV-2 when ketotifen and indomethacin are administered in combination.

### 3.3. Study 3. Cytotoxicity Assay

The results of the cytotoxicity assessments are summarized in Table 5. The 50% cytotoxic concentration (CC_50_) was defined as the compound concentration required for the reduced cell viability by 50%. No cytotoxicity was observed for indomethacin, naproxen and ketotifen at the concentrations tested.

## 4. Discussion

The current findings show that both the combination products ketotifen and indomethacin (SJP-002C) and ketotifen and naproxen reduce viral yield. Compared to ketotifen alone (60% inhibition at EC_50_), an increase in percentage inhibition of SARS-CoV-2 to 90%, 97% and 94% was found when co-administered with 25, 50 and 100 μM indomethacin, respectively, indicating an additive or synergistic effect. The EC_50_ of indomethacin was reduced to <6 μM, equating to ~13-fold greater effect in the presence of ketotifen. Compared to ketotifen alone (60% inhibition at EC_50_), an increase in percentage inhibition of SARS-CoV-2 to 68%, 68% and 92% was found when co-administered with 25, 50 and 100 μM naproxen, respectively. This also indicates an additive or synergistic effect, but the reduction in viral yield was smaller than observed with SJP-002C. Finally, no cytotoxic effects were observed for the concentrations of ketotifen, indomethacin, and naproxen tested.

Scientific evidence supports the observed efficacy of ketotifen in combination with indomethacin (SJP-002C) or naproxen in reducing viral yield. Both naproxen [26,27] and indomethacin [26] have shown—either by computer simulation or in vitro studies—to inhibit viral nucleoprotein involved in viral replication of SARS-CoV-2. Amici et al. [28] described indomethacin’s antiviral activity against SARS-CoV in vitro in Vero E6 cells and in human epithelial lung cells. [29,30,31,32]. In humans, naproxen has been added to oseltamivir and clarithromycin for the treatment of influenza, which resulted in a significantly reduced 30-day mortality, intensive care unit stay, and hospitalization in general [33]. Indomethacin showed efficacy of improved arterial oxygenation in critically ill patients with severe bacterial pneumonia [34] and in adults with respiratory distress syndrome [35]. In relation to the aim of the combination products to reduce excessive immune response, both indomethacin and naproxen have shown to reduce interleukin (IL)-6 in the plasma and synovial fluids of rheumatoid arthritis patients [36], and in another study indomethacin reduced inflammation in Alzheimer patients [37]. These findings suggest that both naproxen and indomethacin may be effective in counteracting the cytokine storm associated with SARS CoV-2 infection [38,39,40].

Ketotifen is approved and marketed in Canada, Europe and Asia for chronic urticaria and childhood asthma for over 20 years, with an excellent safety profile. Mast cells have been implicated in the pathogenesis of viral infections, such as human immunodeficiency virus (HIV)-1, dengue virus, cytomegalovirus, and bovine respiratory syncytial virus [41]. Mast cells can intensify immunological injury through the production of mediators, including tryptase, TNF-α, IL-6, IL-1, and chemokine (C-C motif) ligand 3 (CCL3). Animal studies have shown that ketotifen can reduce excessive inflammation (cytokine storm), and ketotifen has been shown to reduce end organ damage and mortality in mice infected with H5N1 type Influenza A [42]. Ketotifen has been shown in mice infected with H5N1 influenza viral to dramatically reduce lung damage and mortality, even when the antiviral, oseltamivir, was dosed sub-optimally (ketotifen and oseltamivir 100% survival vs. oseltamivir alone 65%) [43]. Another study found that ketotifen reduced vasoactive products and vascular leakage (i.e., an IgG mediated immune response to Dengue virus) in mice [19]. Finally, research also showed that ketotifen can exert a protective effect against NSAID induced GI injury [44,45]. Taken together, these findings support that ketotifen has the potential to reduce excessive inflammation and cytokine storm associated with SARS-CoV-2 infection.

Recent research published as preprint also supports our findings with regard to the efficacy of naproxen and indomethacin in reducing SARS-CoV-2 viral yield. For example, Terrier et al. [46] found that naproxen inhibited viral yield in Vero E6 cells and protected bronchial epithelia against SARS-CoV-2 induced-damage. Building upon the work by Amici et al. [28], Xu et al. [47] conducted antiviral SARS-CoV-2 testing in green monkey kidney Vero E6 cells and found that indomethacin had a direct and potent antiviral activity against SARS CoV-2, without cytotoxicity. In contrast, aspirin, the comparator drug in this study, did not show a significant antiviral effect.

Although the mechanisms underlying the antiviral activity of the combination products ketotifen and indomethacin (SJP-002C) and ketotifen and naproxen against coronavirus infection require further investigation, the results shown herein are promising and warrant further investigation. Future research should confirm our findings in animal models. In addition, future research should confirm whether this observation will modify disease progression in humans, and if ketotifen in combination with indomethacin or naproxen will reduce cytokine storm and subsequent organ damage and mortality rates associated with COVID-19 disease.

## 5. Conclusions

The current findings show that the combination of ketotifen with indomethacin (SJP-002C) or naproxen both significantly reduces viral yield. Compared to ketotifen alone, the combination with indomethacin or naproxen has an additive or synergistic effect. No cytotoxic effects were observed for concentrations of ketotifen, naproxen, and indomethacin tested.

## Figures and Tables

**Table 1:** EC_50_ and CC_50_ data from the CPE assay.

Compound	EC_50_ (μM)	CC_50_ (μM)
Ketotifen	>138.6	138.6
Naproxen	>400	>400
Indomethacin	>400	>400
Remdesivir	2.9	NT

**Table 2:** EC_50_ and percentage inhibition of SARS-CoV-2: 50 μM ketotifen EC_50_ in combination with naproxen.

		Concentration Naproxen (μM)					
Treatment	EC_50_	100	50	25	12.5	6.25	3125
Ketotifen	48.9	-	68	0	0	0	0
Naproxen	>100	0	0	0	21	0	-
Naproxen EC_50_ + 50 μM ketotifen	<6.25	92	68	68	75	60	-

EC_50_ is the effective concentration of product, i.e., the concentration at which virus infection is inhibited by 50 percent.

**Table 3:** EC_50_ and percentage inhibition of SARS-CoV-2: 50 μM ketotifen in combination with indomethacin.

		Concentration Ketotifen (μM)				
Treatment	EC_50_	50	25	12.5	6.25	3125
Ketotifen	47.1	60	0	0	0	0
Ketotifen + 100 μM indomethacin	48.1	94	0	0	37	0
Ketotifen + 50 μM indomethacin	46.5	97	0	0	0	37
Ketotifen + 25 μM indomethacin	42.2	90	0	0	0	0

EC_50_ is the effective concentration of product, i.e., the concentration at which virus infection is inhibited by 50 percent.

**Table 4:** EC_50_ and percentage inhibition of SARS-CoV-2: 100 μM indomethacin in combination with ketotifen.

		Concentration Indomethacin (μM)				
Treatment	EC_50_	100	50	25	12.5	6.25
Indomethacin	100.1	50	0	0	-	-
Indomethacin + 50 μM ketotifen	<6.25	93	83	79	75	60
Indomethacin + 25 μM ketotifen	>100	0	0	0	-	-
Indomethacin + 12.5 μM ketotifen	>100	0	0	0	-	-
Indomethacin + 6.25 μM ketotifen	>100	37	0	0	-	-
Indomethacin + 3.125 μM ketotifen	>100	0	37	0	-	-

EC_50_ is the effective concentration of product, i.e., the concentration at which virus infection is inhibited by 50 percent. - = not assessed.

**Table 5:** Percentage viability at different concentrations of ketotifen, naproxen, and indomethacin.

Concentration (μM)	100	50	25	12.5	6.25	3.13	1.56	0.78	0.39	0.20
Indomethacin (%)	143	143	125	124	124	117	111	106	98	-
Naproxen (%)	107	108	107	107	106	112	96	112	108	-
Ketotifen (%)	-	86	104	122	125	121	109	101	103	110

- = not assessed.

## Data Availability

The data are available from the corresponding author upon reasonable request.
